# The complete chloroplast genome sequence of flowering banana, *Musa ornata*

**DOI:** 10.1080/23802359.2018.1507647

**Published:** 2018-08-29

**Authors:** Jin Liu, Cheng-Wen Gao, Ying-Feng Niu

**Affiliations:** Yunnan Institute of Tropical Crops, Xishuangbanna, China

**Keywords:** *Musa ornata*, flowering banana, chloroplast genome

## Abstract

*Musa ornata* (flowering banana) is one of more than 50 species of banana in the genus *Musa* of the family Musaceae. As sources of resistance to pathogens exist in germplasm, *M. ornata* is one of the possible hybrid parents for ameliorate commercial banana varieties. Herein, we report the complete chloroplast genome of *M. ornata*. The total length of the chloroplast genome is 169,989 bp with 36.82% GC content. The genome consisted of 35,426 bp of a pair of inverted repeat regions (IRs), 10,775 bp of small single copy region (SSC) and 88,362 bp of large single-copy region (LSC). A total of 136 functional genes were annotated, including 113 unique genes comprising 79 protein-coding genes, 4 ribosomal RNA (rRNA) genes and 30 transfer RNA (tRNA) genes. Maximum-likelihood phylogenetic analysis with Musaceae and Zingiberaceae species revealed that *M. ornata* is most closely grouped with *Musa acuminata*.

*Musa ornata* (flowering banana) is one of more than 50 species of banana in the genus *Musa* of the family Musaceae. Based on the basic chromosome number and morphological descriptors, the genus *Musa* has been traditionally divided into four sections, including *Eumusa*, *Rhodochlamys*, *Australimusa* and *Callimusa*, and *M. ornata* is a member of *Rhodochlamys* (Cheesman. 1947). With the development of molecular marker technology, the genus *Musa* has been redivided into two sections, the *Eumusa* and *Rhodochlamys* were merged into the section *Musa*, and the *Australimusa* and *Callimusa* were merged into the section *Callimusa* (Häkkinen. 2013). As sources of resistance to pathogens exist in germplasm, *M. ornata* is one of the possible hybrid parents for ameliorate commercial banana varieties, which has been seriously threatened by the increasing range of fungal, viral, and insect diseases (Novák et al. 2014).

There are poor knowledge on genetic diversity of the genus *Musa*, considering that chloroplast DNA conservativity and its slow rate of nucleotide substitution make chloroplast DNA widely used in plant phylogeny (Sugiura 1995). In this study, we report and characterize the complete chloroplast genome of *M. ornata*.

*Musa ornata* was obtained from CIRAD (Guadeloupe) as rooted plants. Plants were maintained in a greenhouse. Genomic DNA of *M. ornata* was isolated from healthy young leaf tissue (Zhang et al. 1995). Genome sequencing was performed using Roche/454, sequencing libraries were prepared by the GS Titanium library preparation kit (454 Life Sciences, a Rochecompany, Branford, USA). Roche/454 sequencing data were assembled using CLC Genomic Workbench v3.6 (http://www.clcbio.com). The chloroplast genome was annotated using DOGMA (Wyman et al. 2004) with manual correction. The complete chloroplast genome sequence together with gene annotations were submitted to the GenBank with the accession number of MH545183.

The complete size of the *M. ornata* chloroplast genome is 169,989 bp, which includes a pair of inverted repeat regions (IRs) of 35,426 bp separated by a large single-copy region (LSC) of 88,362 bp and a small single copy (SSC) region of 10,775 bp, similar to the previously reported Musaceae chloroplast genomes (Martin et al. 2013; Niu et al. 2018). The size of the *M. ornata* chloroplast genome is larger than *M. acuminate* and *M. balbisiana*. The base composition of the circular chloroplast genome is A (31.43%), G (18.16%), C (18.65%), and T (31.75%). GC content of 36.82% for the whole *M. ornata* chloroplast genome. A total of 113 genes were annotated in *M. ornata* chloroplast genome composed by 79 protein-coding genes, 4 ribosomal RNA (rRNA) genes and 30 transfer RNA (tRNA) genes. The *M. ornata* chloroplast genome has 18 different intron containing genes, two of these genes (ycf3 and clpP) exhibiting two introns and the rest of the genes contain a single intron.

Phylogenetic analysis based on complete plastome sequences was performed using species in Musaceae and Zingiberaceae, and *Oryza sativa* as the outgroup (Figure 1). A total of 10 selected complete plastome sequences were aligned using MAFFT (Katoh and Standley 2013). Maximum-likelihood (ML) analysis was performed using MEGA7 (Kumar et al. 2016) with 1000 bootstrap replicates. The result shows that the phylogenetic relationship of *M. ornata* is close to *M. acuminata*.

**Figure 1.**
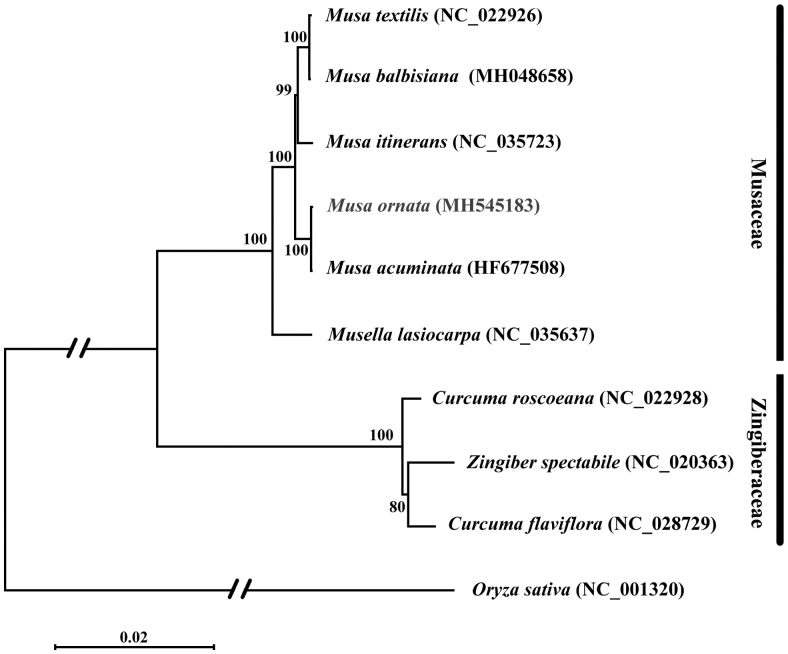
Maximum-likelihood phylogenetic tree of *M. ornata* with 9 species in the order Musaceae and Zingiberaceae based on complete chloroplast genome sequences. Numbers in the nodes are bootstrap values from 1000 replicates. Taxon in red colour is the new genome reported in this study. Bootstrap values are shown above the nodes. The chloroplast genome accession number for tree construction is listed as follows: *Musa ornata* (MH545183), *Musa balbisiana* (MH048658), *Musa textilis* (NC_022926), *Musa itinerans* (NC_035723), *Musa acuminata* (HF677508), *Musella lasiocarpa* (NC_035637), *Curcuma flaviflora* (NC_028729), *Curcuma roscoeana* (NC_022928), *Zingiber spectabile* (NC_020363), *Oryza sativa* (NC_001320).

In conclusion, the complete chloroplast genome sequence of *M. ornata* is decoded for the first time in this study. Moreover, this study will be useful for further phylogenetic and evolutionary analysis for Musaceae.
